# Effects of Different Green Tea Extracts on Chronic Alcohol Induced-Fatty Liver Disease by Ameliorating Oxidative Stress and Inflammation in Mice

**DOI:** 10.1155/2021/5188205

**Published:** 2021-12-29

**Authors:** Bang-Yan Li, Hang-Yu Li, Dan-Dan Zhou, Si-Yu Huang, Min Luo, Ren-You Gan, Qian-Qian Mao, Adila Saimaiti, Ao Shang, Hua-Bin Li

**Affiliations:** ^1^Guangdong Provincial Key Laboratory of Food, Nutrition and Health, Department of Nutrition, School of Public Health, Sun Yat-Sen University, Guangzhou 510080, China; ^2^Research Center for Plants and Human Health, Institute of Urban Agriculture, Chinese Academy of Agricultural Sciences, Chengdu 610213, China

## Abstract

Alcoholic fatty liver disease (AFLD) is a common chronic liver disease and has become a critical global public health problem. Green tea is a popular drink worldwide and contains several bioactive compounds. Different green teas could contain diverse compounds and possess distinct bioactivities. In the present study, the effects of 10 green teas on chronic alcohol induced-fatty liver disease in mice were explored and compared. The results showed that several green teas significantly reduced triacylglycerol levels in serum and liver as well as the aminotransferase activities in mice at a dose of 200 mg/kg, suggesting that they possess hepatoprotective effects. Moreover, several green teas remarkably decreased the expression of cytochrome P450 2E1, the levels of malondialdehyde and 4-hydroxynonenoic acid, and the contents of proinflammatory cytokines, indicating that they could alleviate oxidation damage and inflammation induced by chronic alcohol exposure. In addition, Seven Star Matcha Tea and Selenium-Enriched Matcha Tea could increase glutathione level. Furthermore, the main phytochemical components in green teas were determined and quantified by high-performance liquid chromatography, and the correlation analysis showed that gallic acid, gallocatechin, catechin, chlorogenic acid, and epigallocatechin gallate might at least partially contribute to protective effects on AFLD. In conclusion, Selenium-Enriched Chaoqing Green Tea, Xihu Longjing Tea, Taiping Houkui Tea, and Selenium-Enriched Matcha Tea showed the strongest preventive effects on AFLD. This research also provides the public with new insights about the effects of different green teas on AFLD.

## 1. Introduction

Alcoholic liver disease (ALD) exerts significant morbidity and mortality worldwide [1]. Alcoholic fatty liver disease (AFLD) is the earliest response and primary consequence of chronic excessive alcohol consumption and could develop into more severe pathological stages of ALD, such as alcoholic fibrosis, cirrhosis, and hepatocellular carcinoma [2, 3]. AFLD is characterized by a series of changes, such as hepatic oxidative stress, inflammation, and steatosis, which together contribute to hepatocytes damage [1, 4, 5]. Accordingly, the key to prevent and manage liver injury induced by chronic alcohol exposure is to prevent oxidative stress, inflammation, and steatosis.

Reactive oxygen species (ROS) are required in many important physiological functions, but excessive ROS will react with biological macromolecules in cells, such as lipids, proteins, and nucleic acids and induce various chronic diseases [6, 7]. Oxidative stress is stimulated when the balance of ROS production and antioxidant defense capability is disrupted, which is implicated in certain chronic diseases, such as liver diseases, cardiovascular diseases, and cancers [8–10]. Additionally, several lines of evidence have indicated that the production of excess ROS can be induced by alcohol exposure and metabolism [11]. In fact, the liver is the main organ responsible for metabolizing alcohol [12]. Generally, alcohol is firstly converted into acetaldehyde mainly by alcohol dehydrogenase (ADH), and then aldehyde dehydrogenase (ALDH) metabolizes acetaldehyde to acetic acid [13]. However, during chronic alcohol exposure, cytochrome P450 2E1 (CYP2E1) is induced to replace ADH, playing the major role in alcohol metabolism, which can produce a significant amount of ROS, such as hydrogen peroxide and superoxide anion radicals, resulting in severe oxidative stress [14]. Besides, accumulating studies have revealed that overwhelming oxidative stress induced by alcohol can further lead to the production of lipid peroxides, such as 4-hydroxynonenal (4-HNE) and malondialdehyde (MDA) [15]. Consequently, it has been found that oxidative stress plays a prominent role in the occurrence and progression of AFLD [2]. On the other hand, mounting studies have convinced that inflammation plays an essential role in the initiation and development of AFLD [16, 17]. Furthermore, considerable research has reported that long-term alcohol consumption could influence multiple signaling pathways for lipid metabolism, which increase lipogenesis, inhibit *β*-oxidation of fatty acids, and cause hepatocytes steatosis [18, 19]. Although immediate alcohol abstinence is the most effective therapeutic treatment for AFLD, it is very difficult to carry out for persons with alcohol dependence [20]. Besides, there are no effective drugs for the treatment of AFLD currently. In recent years, early intervention of dietary natural products with strong antioxidant and anti-inflammatory properties has received increasing attention in the prevention and management of AFLD, and more relevant research is urgently needed [3, 21] .

Tea (*Camellia sinensis*) is a widely consumed beverage around the world due to its multiple health benefits [22]. Several lines of studies have proven that green tea imparts numerous biological functions, such as antioxidant and anti-inflammatory activities [23]. In addition, mounting studies have revealed that the various bioactivities of green tea are greatly ascribable to the several bioactive ingredients, such as polyphenols [24]. Moreover, catechins and phenolic acids are the main polyphenols in green tea [25, 26]. Accumulating in vitro evidences have suggested that the catechins in green tea are mainly composed of epigallocatechin gallate (EGCG), epigallocatechin (EGC), epicatechin gallate (ECG), and epicatechin (EC), which have been considered as the major contributor of potent antioxidant property [25, 27]. Several studies have reported the effect of a green tea extract as well as catechins or caffeine from green tea on liver injury induced by alcohol exposure [28–30]. However, different green teas could have distinct effects on AFLD. Thus, in the current study, our goal is to explore and compare the effects of different green teas on AFLD in chronic ethanol-exposed mice, and the main components in green teas are determined and quantified by high-performance liquid chromatography (HPLC). The results could serve the public to select the tea possessing the strongest biological activity on AFLD. Several teas could be also developed into functional foods for the prevention and management of AFLD.

## 2. Materials and Methods

### 2.1. Preparation of Green Tea Extracts

For the preparation of green tea extracts, we depended on the procedures provided in our previous report, including extraction, concentration, and freeze-drying [31]. Firstly, 10 g of green tea was extracted 3 times by using boiling deionized water (100 mL) in water bath (98°C) for 10 min each time, and all extracted solutions were filtered. Secondly, the filtered solution was concentrate, and then completely freeze-dried into a powder for further experiment.  Table 1 displays the detailed information of 10 types of green teas obtained from China.

### 2.2. Measurement of Main Components in Green Teas

The HPLC method was used to determine and quantify the main components in 10 green tea extracts based on our previous report [31], among which the standard compounds were gain from Derick Biotechnology Co., Ltd. (Chengdu, China).

### 2.3. Animals and Experimental Design

Male C57BL/6 J mice (8 weeks old) were obtained from the Guangdong Medical Laboratory Animal Center (Guangzhou, China). All the mice were kept in a specific-pathogen-free (SPF) experimental environment with 12-hour light/dark cycle at 22 ± 0.5°C and had free access to chow diet and water. In addition, all experimental protocols on animals were performed with approve by the “Principles of Care and Use of Laboratory Animals” from the School of Public Health, Sun Yat-Sen University (No. 2019-002; 28 February 2019).

In this study, the Lieber–DeCarli liquid diet purchased from TROPHIC Animal Feed High-tech Co., Ltd. (Nantong, China) was used to establish the AFLD mouse model based on a previous report [32]. This diet contained 4% ethanol (*w/v*) and the ethanol supplied 28% of total calories. In briefly, after one week of acclimatization, all mice were fed Lieber-DeCarli control liquid diet for 5 days to adapt to the liquid diet. After that, according to body weight, the mice were randomly classified into a control group (9 mice) and ethanol-fed groups, which received alcohol adaptation for 6 days. Subsequently, the mice in ethanol-fed groups were further classified into eleven groups (9 mice in each group) including 10 green tea extract supplementary groups and a model group, which were fed with the Lieber-DeCarli ethanol liquid diet. However, the control mice were fed a Lieber-DeCarli control liquid diet during the entire experiment. On the 12^th^ day, mice in all green tea extract supplementary groups were administered with 200 mg/kg.b.w green tea extracts for 4 weeks [33, 34]. Equivalently, about 3.0 g, the green tea was drunk for a person with 60 kg body weight. For another thing, the control and model groups were treated with sterile distilled water (10 mL/kg) for 4 weeks.

At the end of experiment, all mice fasted for 9 hours were anesthetized. The blood samples were obtained from the ophthalmic venous plexus and were centrifuged at the condition of 4,000 × g for 10 min to obtain the serum for biochemical analysis. In addition, the mice were sacrificed, and their liver tissue was collected for further experiments.

### 2.4. Determination of Serum Biochemical Indicators

The liver function biomarkers of alanine transaminase (ALT) and aspartate transaminase (AST) as well as the serum lipid profile of triglyceride (TG) and total cholesterol (TC) were determined with the corresponding kits by using an automated biochemistry analyzer (Roche Diagnostics GmbH, Mannheim, Germany).

### 2.5. Hepatic Histopathological Evaluation by Hematoxylin-Eosin (H&E) Staining

After the mice were sacrificed, the liver samples were immediately fixed in 4% formalin and then embedded in paraffin as well as sectioned. Subsequently, the processed liver tissues were stained using hematoxylin and eosin to assess liver injury induced by chronic alcohol exposure, such as infiltration of inflammatory cells, hepatocyte rearrangement, and lipid accumulation. Finally, a microscope (Leica, Solms, Germany) was used to visualize the histological images. Moreover, using Image-Pro Plus 6.0 analysis software (Media Cybemetics, U.S.A), the vacuolar pixel area within the hepatocytes in each image and tissue pixel area was measured separately (the vacuolar area percentage = vacuolar pixel area/tissue pixel area × 100%).

### 2.6. Analysis of Hepatic Alcohol Metabolism Enzymes

The hepatic samples from all experimental groups were weighed and homogenized in ice-cold 0.9% NaCl to prepare a 10% (w/v) liver tissue homogenate. The activities of ADH and ALDH in liver tissue were determined according to the protocol provided by the kit manufacturer (Nanjing Jiancheng Bioengineering Institute, Nanjing, China) [35, 36].

### 2.7. Analysis of Hepatic Biochemical Indicators

The 200 mg of liver tissue was mixed with 1.8 mL of 0.9% NaCl, and then the mixture was homogenized. Afterward, the liver homogenate was centrifuged (2500 × g, 4°C, and 10 min) to obtain the supernatant, which was used to detect superoxide dismutase (SOD), catalase (CAT), glutathione peroxidase (GSH-Px) activities, and glutathione (GSH) content. The levels of SOD, CAT, GSH-Px, and GSH were measured as previously described by using detection kits purchased from Nanjing Jiancheng Bioengineering Institute (Nanjing, China) [37]. On the other hand, the concentrations of TG, MDA, and total protein were determined by detection kits gained from Apply-gen Technologies Inc. (Beijing, China) [38].

### 2.8. Enzyme-Linked Immunosorbent Assay (ELISA)

The levels of hepatic 4-HNE, CYP2E1, and inflammatory cytokines including interleukin-6 (IL-6) and tumor necrosis factor-*α* (TNF-*α*) were measured by enzyme linked immunosorbent assay (ELISA) based on the instructions (Meimian, Jiangsu, China) [39, 40]. The 0.2 g liver tissue of each mouse was weighed and homogenized thoroughly with 1.8 mL of ice-cold phosphate buffer saline (PBS) and then centrifuged at 3,000 × g for 20 min at 4°C to obtain the supernatant for the assay.

### 2.9. Statistical Analysis

All experimental data were analyzed with the software SPSS 20.0 (IBM SPSS Statistics, IBM Corp, Somers, NY, USA), and the results were expressed as mean ± standard deviation. In addition, a one-way analysis of variance (ANOVA) followed by a least significant difference (LSD) tests was performed to determine statistical difference between experimental groups. When the *p* value <0.05, the result was considered statistically significant. Moreover, graphs were drawn using the software GraphPad Prism 8 (GraphPad Software, La Jolla, CA, USA).

## 3. Results and Discussion

### 3.1. Effects of Green Tea Extracts on Serum Biomarkers

**Figure 1:**
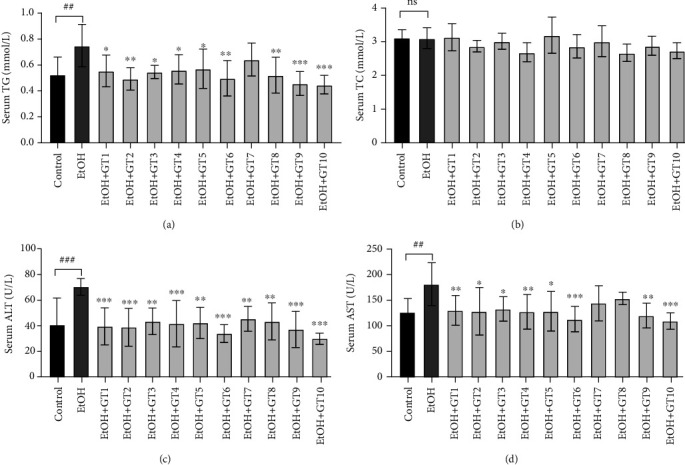
The effects of green teas on serum biomarkers in chronic alcohol-induced fatty liver mice. (a) Serum TG: triacylglycerol. (b) Serum TC: total cholesterol. (c) Serum ALT: alanine aminotransferase. (d) Serum AST: aspartate transaminase. Control: the control group; EtOH: the model group; GT1: Dianqing Tea; GT2: Liping Xiang Tea; GT3: Selenium-Enriched Chaoqing Green Tea; GT4: Xihu Longjing Tea; GT5: Chaoqing Green Tea; GT6: Taiping Houkui Tea; GT7: Jieyang Chaoqing Tea; GT8: Fenggang Zinc-Selenium-enriched Tea; GT9: Seven Star Matcha Tea; GT10: Selenium-Enriched Matcha Tea. *##p* < 0.01; *###p* < 0.001, the model group compared with the control group; ^∗^*p* < 0.05; ^∗∗^*p* < 0.01; ^∗∗∗^*p* < 0.001, the green tea extract supplementary groups compared with the model group.

Increasing evidence has revealed that chronic alcohol consumption could result in lipid metabolism disorders [41, 42]. Additionally, it is well known that the liver is an essential place for modulating lipid metabolism, and the blood is an important transport medium for these metabolites [43]. Thus, the effects of green tea extracts on serum TG and TC levels in chronic alcohol-induced fatty liver mice were studied as shown in Figures 1(a) and 1(b). The serum TG level in the model group was significantly elevated in comparison with the control group (*p* < 0.01). Moreover, the increased serum TG level induced by chronic alcohol exposure was effectively restored by the supplementation of most green tea extracts, except for Jieyang Chaoqing Tea (GT7). On the other hand, there was no marked difference in serum TC level among all groups (*p* > 0.05).

It has been demonstrated that increased serum ALT and AST levels are reliable indicator of liver injury [44, 45]. Seen from the Figures 1(c) and 1(d), the model group exhibited a significant elevation in serum ALT (*p* < 0.001) and AST (*p* < 0.01) activities compared with the control group. Consistent with our results, previous reports have revealed that an increase in serum AST and ALT activities was observed in mice and rats exposed to alcohol [46, 47]. In the present study, treatment with most green tea extracts effectively ameliorated liver injury by inhibiting the elevated ALT and AST activities induced by alcohol consumption, while supplementation with Jieyang Chaoqing Tea (GT7) and Fenggang Zinc-Selenium-enriched Tea (GT8) only reduced AST activity. The results indicated that most green tea extracts possessed preventive effects on AFLD induced by chronic alcohol exposure. It has been reported that some green tea extracts, such as Liping Xiang Tea and Seven Star Matcha Tea, could protect acute alcohol consumption-induced liver injury by reducing the activities of AST and/or ALT [31]. Furthermore, in agreement to our finding, a previous animal study showed that an increased ALT activity caused by alcohol was inhibited by the treatment of green tea extracts in rats [28].

### 3.2. Effects of Green Tea Extracts on Alcohol Metabolism

**Figure 2:**
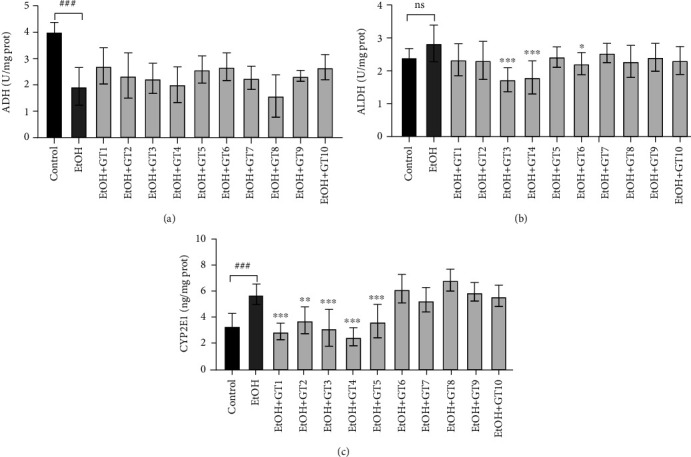
The effects of green teas on alcohol metabolism in chronic alcohol-induced fatty liver mice. (a) ADH: alcohol dehydrogenase; (b) ALDH: aldehyde dehydrogenase; (c) CYP2E1: cytochrome P450 2E1. Control: the control group; EtOH: the model group; GT1: Dianqing Tea; GT2: Liping Xiang Tea; GT3: Selenium-Enriched Chaoqing Green Tea; GT4: Xihu Longjing Tea; GT5: Chaoqing Green Tea; GT6: Taiping Houkui Tea; GT7: Jieyang Chaoqing Tea; GT8: Fenggang Zinc-Selenium-enriched Tea; GT9: Seven Star Matcha Tea; GT10: Selenium-Enriched Matcha Tea. *###p* < 0.001, the model group compared with the control group; ^∗^*p* < 0.05; ^∗∗^*p* < 0.01; ^∗∗∗^*p* < 0.001, the green tea extract supplementary groups compared with the model group.

The effects of green teas on alcohol metabolism in chronic alcohol-induced fatty liver mice are presented in Figure 2. In the present study, we found that the activity of ADH was significantly inhibited, and the CYP2E1 enzymes were remarkably induced by chronic alcohol exposure. Although an increased tendency in ALDH activity was shown in the model group compared with the control group, there was no significant difference. Under normal conditions, alcohol metabolism consists of two steps, where ethanol is initially converted to acetaldehyde via ADH, and then acetaldehyde is further oxidized to acetic acid by ALDH [48, 49]. Accumulating evidence has demonstrated that ADH would be replaced by CYP2E1 enzymes in the liver under long-term alcohol exposure, which could result in severe oxidative stress [2, 50].

As shown in Figure 2(c), the elevated CYP2E1 enzymes induced by chronic alcohol exposure were effectively restored by supplementation with Dianqing Tea (GT1), Liping Xiang Tea (GT2), Selenium-Enriched Chaoqing Green Tea (GT3), Xihu Longjing Tea (GT4), and Chaoqing Green Tea (GT5). The results suggested that these teas could ameliorate AFLD caused by alcohol through inhibiting CYP2E1 enzymes. Consistent with our results, a previous in vitro study revealed that intervention with catechin and caffeine from green tea extract could obviously restrain the overexpression of CYP2E1 in the HepG2 cell model [51]. In addition, a previous animal experiment reported that catechins derived from green tea extracts decreased the expression of oxidative stress-derived enzymes, such as CYP2E1 [28]. According to the results, all green tea extracts insignificantly influenced the activity of ADH compared with the model group, while the ALDH activity was markedly inhibited by the supplementation of Selenium-Enriched Chaoqing Green Tea (GT3), Xihu Longjing Tea (GT4) ,and Taiping Houkui Tea (GT6). It illustrated that these green teas inhibited the conversion of acetaldehyde to acetic acid followed by an increase in the acetaldehyde concentration, which would aggravate health damage [52, 53]. Moreover, the results indicated the side effects of these green teas on the alcohol drinking.

### 3.3. Effects of Green Tea Extracts on Body Weight and Liver Lipid Profiles

**Figure 3:**
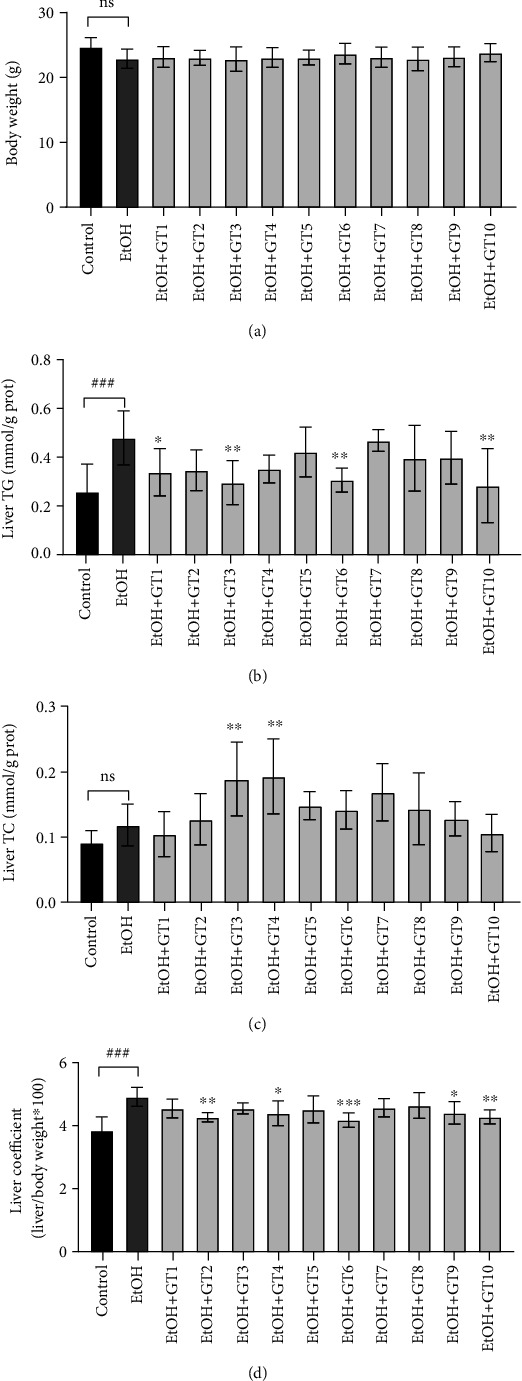
The effects of green teas on liver lipid profiles in chronic alcohol-induced fatty liver mice. (a) Body weight. (b) Liver TG: triacylglycerol. (c) Liver TC: total cholesterol. (d) liver coefficient. Control: the control group; EtOH: the model group; GT1: Dianqing Tea; GT2: Liping Xiang Tea; GT3: Selenium-Enriched Chaoqing Green Tea; GT4: Xihu Longjing Tea; GT5: Chaoqing Green Tea; GT6: Taiping Houkui Tea; GT7: Jieyang Chaoqing Tea; GT8: Fenggang Zinc-Selenium-enriched Tea; GT9: Seven Star Matcha Tea; GT10: Selenium-Enriched Matcha Tea. *###p* < 0.001, the model group compared with the control group; ^∗^*p* < 0.05; ^∗∗^*p* < 0.01; ^∗∗∗^*p* < 0.001, the green tea extract supplementary groups compared with the model group.

In the current study, the liquid feed intake volume of the mice in control and all green tea extract supplementary groups were determined based on that of the model group in the previous day to control the energy intake to be equal. Thus, it was observed that there was no significant difference in body weight among mice of all groups at the end of experiment (Figure 3(a)).

As displayed in Figures 3(b)–3(d), compared with the control group, hepatic TG concentration and liver coefficient were remarkably elevated in the model group, but there was no significant change in the hepatic TC content. The results were agreement with previous studies in which reported long-term excessive consumption of alcohol could result in abnormal metabolism of hepatic TG and liver steatosis [54, 55]. Seen from our results, the increased hepatic TG level induced by chronic alcohol exposure was effectively reduced by the supplementation of Dianqing Tea (GT1), Selenium-Enriched Chaoqing Green Tea (GT3), Taiping Houkui Tea (GT6), and Selenium-Enriched Matcha Tea (GT10), indicating that these green teas could modulate abnormal hepatic TG accumulation in chronic alcohol-induced mice. Meanwhile, the abnormity of liver coefficient caused by alcohol was obviously restored by the treatment of Liping Xiang Tea (GT2), Xihu Longjing Tea (GT4), Taiping Houkui Tea (GT6), Seven Star Matcha Tea (GT9), and Selenium-Enriched Matcha Tea (GT10). In addition, the supplementation of Selenium-Enriched Chaoqing Green Tea (GT3) and Xihu Longjing Tea (GT4) could obviously increase hepatic TC content, which is different from the results of serum. This could be because the liver is the main organ for the synthesis and storage of total cholesterol.

### 3.4. Histopathological Evaluation

**Figure 4:**
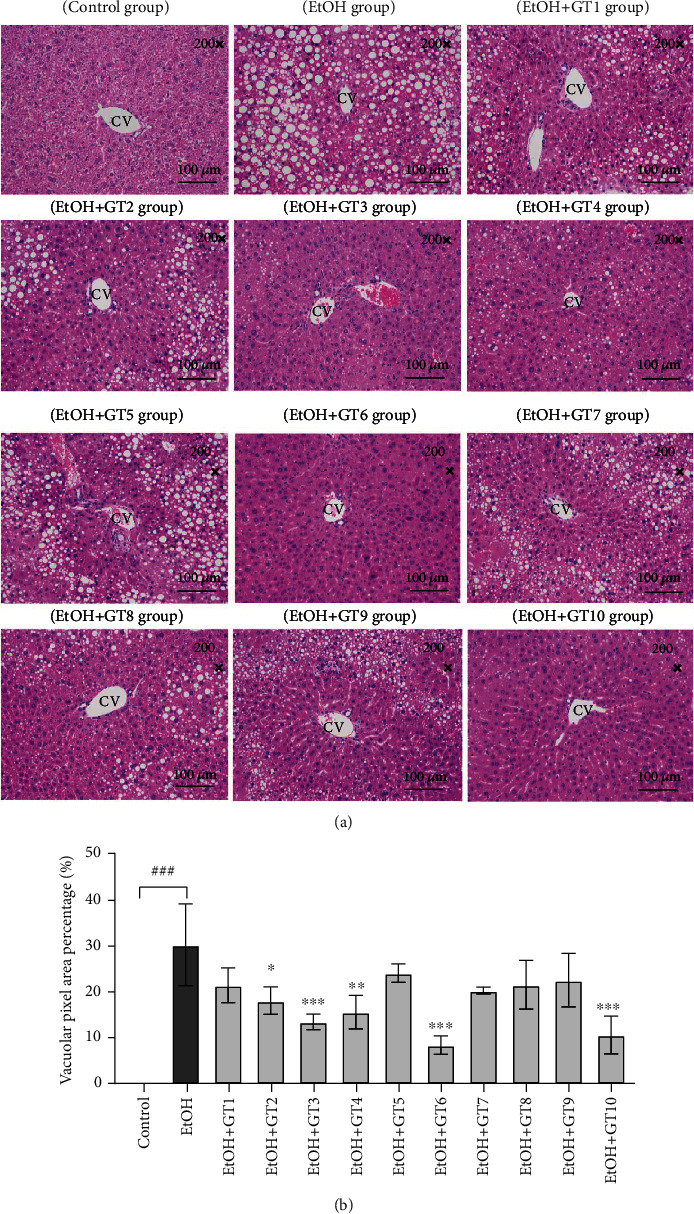
The histopathological evaluation for all experiment groups. (a) The photomicrographs of hematoxylin and eosin (H&E) stained for liver sections (magnification: 200; scale bar: 100 *μ*m; CV: central venous). (b) The vacuolar pixel area percentage. Control: the control group; EtOH: the model group; GT1: Dianqing Tea; GT2: Liping Xiang Tea; GT3: Selenium-Enriched Chaoqing Green Tea; GT4: Xihu Longjing Tea; GT5: Chaoqing Green Tea; GT6: Taiping Houkui Tea; GT7: Jieyang Chaoqing Tea; GT8: Fenggang Zinc-Selenium-enriched Tea; GT9: Seven Star Matcha Tea; GT10: Selenium-Enriched Matcha Tea. *###p* < 0.001, the model group compared with the control group; ^∗^*p* < 0.05; ^∗∗^*p* < 0.01; ^∗∗∗^*p* < 0.001, the green tea extract supplementary groups compared with the model group.

To further confirm the preventive effect of green teas on AFLD induced by chronic ethanol exposure, H&E staining was used to observe the histopathological morphologies of the liver sections. As displayed in Figure 4, compared with the normal group mice, the liver of chronic alcohol-treated mice showed remarkable deposition with large numbers of medium and small lipid droplets in the parenchyma cells. However, supplementation with certain green teas reduced lipid droplets in hepatic cells and attenuated the liver injury induced by ethanol, especially Selenium-Enriched Chaoqing Green Tea (GT3), Xihu Longjing Tea (GT4), Taiping Houkui Tea (GT6), and Selenium-Enriched Matcha Tea (GT10). In addition, it should also be point out that the degree of inflammatory damage in ethanol-fed mice was less severe in this study, and the inflammatory damage of ethanol-fed mice was not observed by hepatic histopathological evaluation; although, the release of proinflammatory cytokines was obvious (see latter section).

### 3.5. Effects of Green Tea Extracts on Oxidative Damage

**Figure 5:**
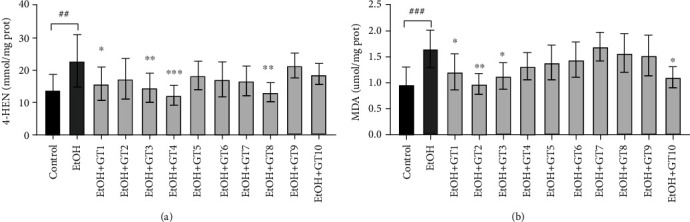
The effects of green teas on oxidative stress damage in chronic alcohol-induced fatty liver mice. (a) 4-HNE: 4-hydroxynonenoic acid; (b) MDA: malondialdehyde. Control: the control group; EtOH: the model group; GT1: Dianqing Tea; GT2: Liping Xiang Tea; GT3: Selenium-Enriched Chaoqing Green Tea; GT4: Xihu Longjing Tea; GT5: Chaoqing Green Tea; GT6: Taiping Houkui Tea; GT7: Jieyang Chaoqing Tea; GT8: Fenggang Zinc-Selenium-enriched Tea; GT9: Seven Star Matcha Tea; GT10: Selenium-Enriched Matcha Tea. *##p* < 0.01; *###p* < 0.001, the model group compared with the control group; ^∗^*p* < 0.05; ^∗∗^*p* < 0.01; ^∗∗∗^*p* < 0.001, the green tea extract supplementary groups compared with the model group.

Recent work has revealed that CYP2E1 enzymes induced by chronic alcohol exposure can result in producing excessive ROS, which are correlated with lipid peroxidation [56]. Seen from the Figure 5, we observed that the levels of 4-HNE and MDA were significantly increased by chronic alcohol exposure compared with the control group. A previous study revealed that oxidative stress in transgenic mice overexpressing CYP2E1 was enhanced [57]. However, it has been reported that alcohol-medicated lipid peroxidation was significantly blocked by CYP2E1 deletion, and the liver damage was relieved [58]. In the present work, Dianqing Tea (GT1), Selenium-Enriched Chaoqing Green Tea (GT3) and Xihu Longjing Tea (GT4), and Fenggang Zinc-Selenium-enriched Tea (GT8) effectively alleviated liver lipid peroxidation by decreasing the 4-HNE level. The results were consistent with the fact that several teas could significantly decrease chronic alcohol-induced CYP2E1 enzymes level. In addition, the level of MDA was obviously reduced by Dianqing Tea (GT1), Liping Xiang Tea (GT2), Selenium-Enriched Chaoqing Green Tea (GT3), and Selenium-Enriched Matcha Tea (GT10). The results indicated that these teas possessed preventive effects on lipid peroxidation of liver caused by ethanol.

### 3.6. Effects of Green Tea Extracts on Antioxidant Capacity

**Figure 6:**
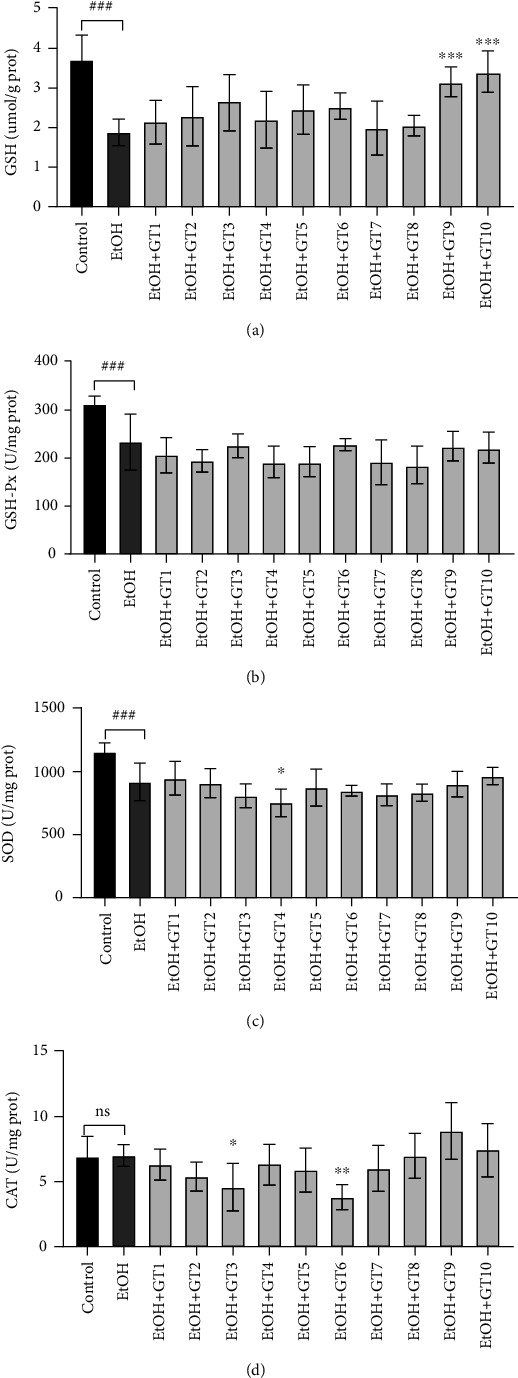
The effects of green teas on oxidant ability in chronic alcohol-induced fatty liver mice. (a) GSH: glutathione. (b) GSH-Px: glutathione peroxidase. (c) SOD: superoxide dismutase. (d) CAT: catalase. Control: the control group; EtOH: the model group; GT1: Dianqing Tea; GT2: Liping Xiang Tea; GT3: Selenium-Enriched Chaoqing Green Tea; GT4: Xihu Longjing Tea; GT5: Chaoqing Green Tea; GT6: Taiping Houkui Tea; GT7: Jieyang Chaoqing Tea; GT8: Fenggang Zinc-Selenium-enriched Tea; GT9: Seven Star Matcha Tea; GT10: Selenium-Enriched Matcha Tea. *###p* < 0.001, the model group compared with the control group; ^∗^*p* < 0.05; ^∗∗^*p* < 0.01; ^∗∗∗^*p* < 0.001, the green tea extract supplementary groups compared with the model group.

It has been identified that alcohol exposure will significantly decrease in vivo antioxidant capacity [59]. In the present study (Figure 6), chronic alcohol consumption resulted in a remarkable reduction in hepatic GSH content as well as the activities of GSH-Px and SOD compared with the control group, which indicated a significant inhibition in the antioxidant capacity of the liver. A previous animal research reported that the activities of enzymatic antioxidants (GSH-Px and SOD) were inhibited, and the content of nonenzymatic antioxidants (GSH) was reduced in acute alcohol-induced liver damage mice [60]. In addition, compared with the control group, there was no statistical difference in the change in CAT activity induced by chronic alcohol exposure.

Accumulating findings have demonstrated that green tea contained abundant phenolic acids and catechins, especially EGCG, which were responsible for the strong antioxidant properties of green tea [25, 61]. In addition, it has been reported that green tea had strong in vitro antioxidant ability [62]. In this study, we found that only Seven Star Matcha Tea (GT9) and Selenium-Enriched Matcha Tea (GT10) significantly elevated the GSH content, while the intervention of most green tea extracts did not influence the activities of SOD, CAT, and GSH-Px (Figure 6). Moreover, Xihu Longjing Tea (GT4) obviously inhibited the SOD activity and Selenium-Enriched Chaoqing Green Tea (GT3) and Taiping Houkui Tea (GT6) remarkably decreased CAT activity, which illustrated that these teas could reduce the in vivo antioxidant ability in chronic alcohol-induced fatty liver mice.

### 3.7. Effects of Green Tea Extracts on Inflammation Levels

**Figure 7:**
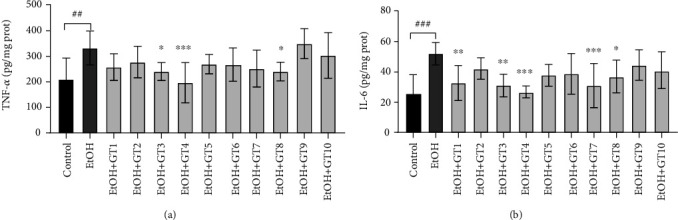
The effects of green teas on inflammation in chronic alcohol-induced fatty liver mice. (a) TNF-*α*: tumor necrosis factor-*α*. (b) IL-6: interleukin-6. Control: the control group; EtOH: the model group; GT1: Dianqing Tea; GT2: Liping Xiang Tea; GT3: Selenium-Enriched Chaoqing Green Tea; GT4: Xihu Longjing Tea; GT5: Chaoqing Green Tea; GT6: Taiping Houkui Tea; GT7: Jieyang Chaoqing Tea; GT8: Fenggang Zinc-Selenium-enriched Tea; GT9: Seven Star Matcha Tea; GT10: Selenium-Enriched Matcha Tea. *##p* < 0.01; *###p* < 0.001, the model group compared with the control group; ^∗^*p* < 0.05; ^∗∗^*p* < 0.01; ^∗∗∗^*p* < 0.001, the green tea extract supplementary groups compared with the model group.

The available evidence revealed that alcohol exposure results in inflammation [63]. In this study, we observed that chronic alcohol consumption induced a significant increase in the levels of IL-6 and TNF-*α* in liver compared to the control group. As shown in Figure 7, Selenium-Enriched Chaoqing Green Tea (GT3), Xihu Longjing Tea (GT4), and Fenggang Zinc-Selenium-enriched Tea (GT8) effectively ameliorated inflammation induced by alcohol through decreasing IL-6 and TNF-*α* levels. Moreover, the IL-6 level was reduced by Dianqing Tea (GT1) and Jieyang Chaoqing Tea (GT7). Consistent with our results, a previous study reported that catechins from tea could effectively inhibited proinflammatory signal pathways and relieve inflammation [28].

### 3.8. Relation of Biochemical Indicators and Components of Green Teas

**Figure 8:**
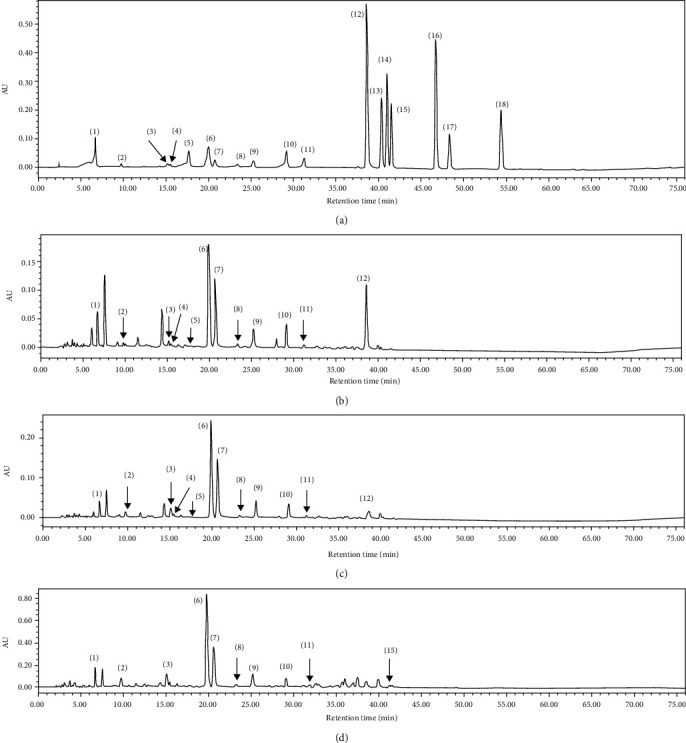
The HPLC chromatograms of the standard compounds. (a) Xihu Longjing Tea. (b) Taiping Houkui Tea. (c) Selenium-Enriched Matcha Tea. (d) Under 254 nm. 1, gallic acid; 2, gallocatechin; 3, epigallocatechin; 4, catechin; 5, chlorogenic acid; 6, caffeine; 7, epigallo-catechin gallate; 8, epicatechin; 9, gallocatechin gallate; 10, epicatechin gallate; 11, catechin gallate; 12, ellagic acid; 13, myricetin; 14, quercitrin; 15, astragalin; 16, quercetin; 17, theaflavin; 18, kaempferol.

In the current study, the main components in 10 green tea extracts were detected and quantified by HPLC. The chromatograms of standard components, Xihu Longjing Tea (GT4), Taiping Houkui Tea (GT6), and Selenium-Enriched Matcha Tea (GT10) under 254 nm wavelength, are displayed in Figure 8. Additionally, the results of main components in 10 green teas are shown in Table 2.

Based on our results, 13 components in 10 green tea extracts have been determined and quantified, including 8 kinds of catechins and 5 other active ingredients (gallic acid, chlorogenic acid, caffeine, ellagic acid, and astragalin). The results showed that catechins are the most abundant phytochemicals in these green tea extracts, but the content of catechins in theses green teas varied greatly. We observed that EGCG was the richest catechin with a range from 13.462 ± 0.206 to 62.164 ± 0.298 mg/g DW (dry weight of tea). In addition, all green teas were found with high contents of caffeine, with a range of 25.973 ± 0.054 to 46.218 ± 0.377 mg/g DW. However, myricetin, quercitrin, quercetin, theaflavin, and kaempferol have not been identified.

The relationship between the main components in green teas and the biochemical indicators was analyzed in the present study. According to the results, some phytochemicals in green tea were associated with serum TC and TG concentrations as well as serum aminotransferase activity (such as AST). For example, a middle negative (*p* < 0.05) correlation has been found between the content of gallocatechin and the serum TC level, and the *R*^2^ value was 0.6018. However, the content of catechin was positively (*p* < 0.05) related to serum TG level, and the *R*^2^ value was 0.3618. In addition, we observed that the contents of gallic acid and EGCG were positively (*p* < 0.05) associated with the activity of AST, and the *R*^2^ values were 0.4972 and 0.4967, respectively. Moreover, the results showed that the content of gallic acid was negatively (*p* < 0.05) related to the activity of ADH, and the *R*^2^ value was 0.5194.

**Figure 9:**
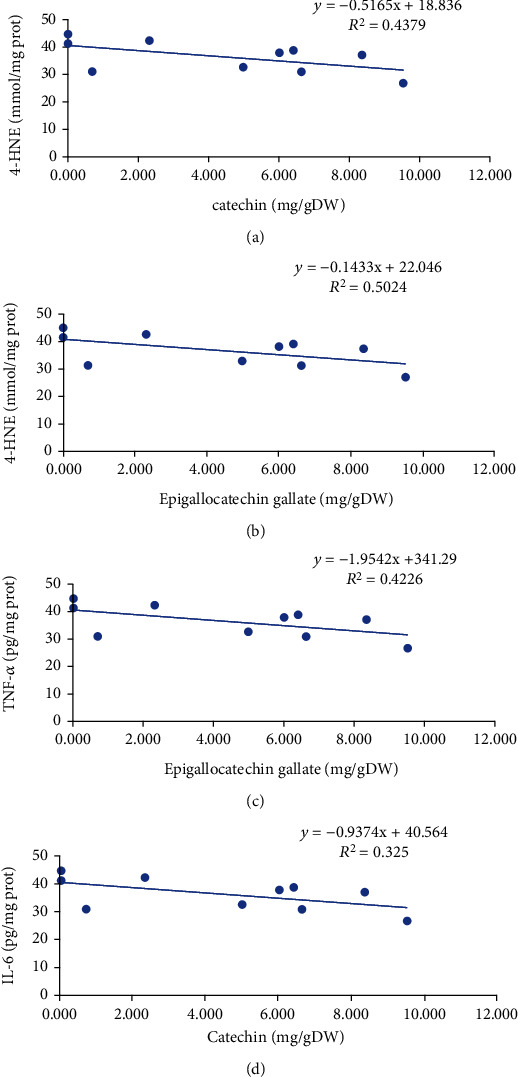
The relationships between biochemical indicators and phytochemical components of green tea. (a) Correlation between the level of 4-HNE and catechin. (b) Correlation between the level of 4-HNE and epigallocatechin gallate. (c) Correlation between the concentration of TNF-*α* in the liver and epigallocatechin gallate. (d) Correlation between the concentration of IL-6 in serum and catechin. 4-HNE: 4-hydroxynonenoic acid; TNF-*α*: tumor necrosis factor-*α*; IL-6: interleukin-6.

The contents of gallic acid, catechin, chlorogenic acid, and EGCG were all negatively (*p* < 0.05) related to the level of 4-HNE, and the *R*^2^ values were 0.3433, 0.4379, 0.4317, and 0.5024, respectively (Figures 9(a) and 9(b)). The results indicate that gallic acid, catechin, chlorogenic acid, and EGCG in green tea extracts might be closely related to ameliorating chronic alcohol-induced lipid peroxidation damage. In addition, the concentration of GSH showed a significant negative (*p* < 0.05) correlation with the contents of catechin, chlorogenic acid, and EGCG, and the *R*^2^ values were 0.6031, 0.4225, and 0.5726, respectively. Moreover, a weak negative (*p* < 0.05) correlation was observed between the activity of GSH-Px and the content of catechin, and the *R*^2^ value was 0.4541. Besides, the content of catechin was negatively (*p* < 0.05) associated with the activity of SOD, and the *R*^2^ value was 0.367. The results suggest that catechin, chlorogenic acid, and EGCG might at least partially inhibit the antioxidant activity of green tea in vivo.

The correlations between these main components and the inflammatory cytokines (TNF-*α* and IL-6) were also analyzed (Figures 9(c) and 9(d)). The results showed that the concentrations of catechin, chlorogenic acid, and EGCG were negatively (*p* < 0.05) correlated with the level of TNF-*α*, and the *R*^2^ values were 0.5438, 0.4167, and 0.4226, respectively. Furthermore, the level of IL-6 showed an obvious negative (*p* < 0.05) correlation with the contents of catechin and chlorogenic acid, and the *R*^2^ values were 0.325 and 0.5724, respectively. These results indicate that catechin, chlorogenic acid, and EGCG in green tea might be related to anti-inflammatory property.

## 4. Conclusions

The effects of 10 green teas on mice with AFLD caused by chronic alcohol exposure and the correlation between their main components and AFLD were studied and compared. The results revealed that most green teas significantly reduced the levels of TG and the aminotransferase activities (such as AST and ALT), indicating that they possess hepatoprotective effects. In addition, certain green teas remarkably decreased the expression of CYP2E1, the levels of MDA and 4-HNE, and the contents of TNF-*α* and IL-6, suggesting that they could relieve oxidation damage and inflammation induced by chronic ethanol consumption. Moreover, Seven Star Matcha Tea and Selenium-Enriched Matcha Tea could increase GSH content. On the other hand, the most abundant ingredients are catechins and caffeine among the 10 green teas. Moreover, the correlation analysis showed that gallic acid, gallocatechin, catechin, chlorogenic acid, and EGCG might at least partially contribute to preventive effects on AFLD. On the other hand, some green teas could increase hepatic TC content and decrease SOD, CAT, and ALDH activities, which could result in some adverse effects. In previous review paper [62], the adverse effects of various teas have been summarized in detail, most studies have focused on nonalcoholic fatty liver disease and normal persons, and no report about green teas against ALD could be found. In the future, a special attention should be paid to the adverse effects of teas (including green tea) and their components against ALD. In total, four teas, including Selenium-Enriched Chaoqing Green Tea, Xihu Longjing Tea, Taiping Houkui Tea, and Selenium-Enriched Matcha Tea, showed the stronger preventive effects on AFLD than other six teas, but the difference was very less among four teas. These four green teas could be developed into functional foods for the prevention and management of AFLD. Furthermore, the results provide the public and nutritionists with new insights about the effects of green teas on AFLD.

## Figures and Tables

**Table 1:** The detailed information of 10 green teas from China.

Number	Name	Production place
GT1	Dianqing Tea	Kunming, Yunnan
GT2	Liping Xiang Tea	Liping, Guizhou
GT3	Selenium-enriched Chaoqing Green Tea	Enshi, Hubei
GT4	Xihu Longjing Tea	Hangzhou, Zhejiang
GT5	Chaoqing Green Tea	Yichang, Hubei
GT6	Taiping Houkui Tea	Huangshan, Anhui
GT7	Jieyang Chaoqing Tea	Jieyang, Guangdong
GT8	Fenggang Zinc-Selenium-Enriched Tea	Guiyang, Guizhou
GT9	Seven Star Matcha Tea	Shaoxing, Zhejiang
GT10	Selenium-enriched Matcha Tea	Enshi, Hubei

**(a):** 

Main phytochemicals	Dianqing Tea	Liping Xiang Tea	Selenium-Enriched Chaoqing Green Tea	Xihu Longjing Tea	Chaoqing Green Tea
Gallic acid	1.698 ± 0.027	1.588 ± 0.029	1.575 ± 0.035	1.657 ± 0.064	0.683 ± 0.086
Gallocatechin	4.577 ± 0.257	7.901 ± 0.120	6.411 ± 0.416	8.050 ± 0.060	4.941 ± 0.180
Epigallocatechin	13.573 ± 0.253	23.390 ± 0.029	8.654 ± 0.397	19.028 ± 0.040	9.339 ± 0.281
Catechin	4.985 ± 0.197	2.313 ± 0.003	0.690 ± 0.014	9.533 ± 0178	6.010 ± 0.062
Chlorogenic acid	1.230 ± 0.003	—	0.830 ± 0.001	0.804 ± 0.000	—
Caffeine	33.925 ± 0.172	27.664 ± 0.174	31.137 ± 0.228	39.859 ± 0.234	32.766 ± 0.327
Epigallocatechin gallate	53.368 ± 0.463	37.705 ± 0.193	43.304 ± 0.889	34.741 ± 0.284	40.110 ± 1.120
Epicatechin	8.901 ± 0.153	2.665 ± 0.056	5.517 ± 0.076	5.295 ± 0.104	5.249 ± 0.180
Gallocatechin gallate	16.895 ± 0.344	6.222 ± 0.119	—	15.134 ± 1.029	13.950 ± 1.056
Epicatechin gallate	17.649 ± 0.537	3.329 ± 0.020	6.340 ± 0.158	17.188 ± 0.052	17.683 ± 0.030
Catechin gallate	3.475 ± 0.039	0.391 ± 0.009	—	1.747 ± 0.031	2.244 ± 0.041
Ellagic acid	0.778 ± 0.023	—	—	0.950 ± 0.004	—
Myricetin	—	—	—	—	—
Quercitrin	—	—	—	—	—
Astragalin	0.616 ± 0.007	2.702 ± 0.039	—	—	1.053 ± 0.006
Quercetin	—	—	—	—	—
Theaflavin	—	—	—	—	—
Kaempferol	—	—	—	—	—

**(b):** 

Main phytochemicals	Taiping Houkui Tea	Jieyang Chaoqing Tea	Fenggang Zinc-Selenium-Enriched Tea	Seven Star Matcha Tea	Selenium-Enriched Matcha Tea
Gallic acid	0.929 ± 0.043	1.84 ± 0.023	2.335 ± 0.053	1.298 ± 0.031	1.411 ± 0.027
Gallocatechin	9.721 ± 0.076	6.109 ± 0.030	8.667 ± 1.164	5.736 ± 0.075	7.409 ± 0.091
Epigallocatechin	17.590 ± 0.201	9.151 ± 0.219	13.039 ± 0.074	20.865 ± 0.073	9.425 ± 0.050
Catechin	6.407 ± 0.447	6.635 ± 0.143	8.362 ± 0.214	—	—
Chlorogenic acid	0.862 ± 0.005	1.095 ± 0.005	0.819 ± 0.000	—	—
Caffeine	46.218 ± 0.377	25.973 ± 0.054	37.421 ± 0.445	28.715 ± 0.158	29.340 ± 0.270
Epigallocatechin gallate	38.205 ± 0.873	38.687 ± 0.198	62.164 ± 0.298	13.462 ± 0.206	24.264 ± 0.743
Epicatechin	5.268 ± 0.112	6.307 ± 0.114	7.125 ± 0.060	7.421 ± 0.133	3.379 ± 0.052
Gallocatechin gallate	16.140 ± 0.574	9.724 ± 0.147	16.612 ± 0.153	—	9.030 ± 0.069
Epicatechin gallate	16.259 ± 0.180	8.073 ± 0.025	11.411 ± 0.158	8.727 ± 0.079	9.378 ± 0.038
Catechin gallate	1.577 ± 0.024	1.584 ± 0.109	3.056 ± 0.090	0.375 ± 0.001	4.844 ± 0.109
Ellagic acid	0.893 ± 0.044	—	2.069 ± 0.019	—	0.241 ± 0.043
Myricetin	—	—	—	—	—
Quercitrin	—	—	—	—	—
Astragalin	—	2.839 ± 0.024	—	1.823 ± 0.094	—
Quercetin	—	—	—	—	—
Theaflavin	—	—	—	—	—
Kaempferol	—	—	—	—	—

Note. -: not determined; DW: dry weight of tea.

## Data Availability

The data is kept in School of Public Health, Sun Yat-Sen University.
